# Inter- and intra-specific variation in drought sensitivity in *Abies spec*. and its relation to wood density and growth traits

**DOI:** 10.1016/j.agrformet.2015.08.268

**Published:** 2015-12-15

**Authors:** Jan-Peter George, Silvio Schueler, Sandra Karanitsch-Ackerl, Konrad Mayer, Raphael T. Klumpp, Michael Grabner

**Affiliations:** a Federal Research and Training Centre for Forests, Natural Hazards and Landscape (BFW), Department of Forest Genetics, Seckendorff-Gudent-Weg 8, 1131 Vienna, Austria; b University of Natural Resources and Life Sciences (BOKU), Institute of Wood Science and Technology, Konrad-Lorenz-Straβe 24, 3430 Tulln an der Donau, Austria; c University of Natural Resources and Life Sciences (BOKU), Institute of Silviculture, Peter-Jordan-Straβe 82, 1190 Vienna, Austria

**Keywords:** Conifer, *Silver fir*, Drought response, Wood density, Growth reduction

## Abstract

Understanding drought sensitivity of tree species and its intra-specific variation is required to estimate the effects of climate change on forest productivity, carbon sequestration and tree mortality as well as to develop adaptive forest management measures. Here, we studied the variation of drought reaction of six European *Abies* species and ten provenances of *Abies alba* planted in the drought prone eastern Austria. Tree-ring and X-ray densitometry data were used to generate early- and latewood measures for ring width and wood density. Moreover, the drought reaction of species and provenances within six distinct drought events between 1970 and 2011, as identified by the standardized precipitation index, was determined by four drought response measures. The mean reaction of species and provenances to drought events was strongly affected by the seasonal occurrence of the drought: a short, strong drought at the beginning of the growing season resulted in growth reductions up to 50%, while droughts at the end of the growing season did not affect annual increment. Wood properties and drought response measures showed significant variation among *Abies* species as well as among *A. alba* provenances. Whereas *A. alba* provenances explained significant parts in the variation of ring width measures, the *Abies* species explained significant parts in the variation of wood density parameters. A consistent pattern in drought response across the six drought events was observed only at the inter-specific level, where *A. nordmanniana* showed the highest resistance and *A. cephalonica* showed the best recovery after drought. In contrast, differences in drought reaction among provenances were only found for the milder drought events in 1986, 1990, 1993 and 2000 and the ranking of provenances varied at each drought event. This indicates that genetic variation in drought response within *A. alba* is more limited than among *Abies* species. Low correlations between wood density parameters and drought response measures suggest that wood density is a poor predictor of drought sensitivity in *Abies spec*.

## 1 Introduction

Terrestrial plants have developed various strategies to avoid or reduce drought induced stress. Such strategies encompass, amongst others, anatomical or physiological adjustments like reduction of leaf area (Le Dantec et al., 2000), reallocation of biomass from the crown to stem and roots (DeLucia et al., 2000), stomatal closure (Sperry, 2000) or altering gene expression patterns of proteins (Wang et al., 2003). One important milestone in the evolution of terrestrial plants had been the development of woody tissue, especially of xylem conduits, which allows them to transport water along a soil-plant-atmosphere continuum to greater heights efficiently (Breda et al., 2006; Sperry et al., 2008). This mechanism also bears risks, especially when soil-water is limited under strong evaporative demand: if the water flow within the xylem conduits is disrupted cavitation may occur causing irreversible damages to the water transport system (Choat et al., 2012). A tree’s vulnerability to cavitation depends on many factors such as the pore diameter in conduit walls (i.e. the greater the diameter, the higher the vulnerability) (Zimmermann, 1978) and conduit wall reinforcement (i.e. the greater the reinforcement, the lower the vulnerability) (Hacke et al., 2001). However, to measure these anatomical characteristics and to estimate a tree’s vulnerability to cavitation requires sophisticated preparation and measuring techniques and the ultimate destruction of the given sample. Thus, less time-consuming measures of wood traits were tested as indicators of cavitation- and drought-sensitivity. In Norway spruce (*Picea abies* L.), Rosner et al. (2014) found a negative relationship between wood density and the pressure potential necessary to induce 50% (P_50_) and 88% (P_88_) loss of hydraulic conductivity, respectively. Similar strong relationships were also found for Douglas-fir (*Pseudotsuga menziesii* (Mirb.) Franco) (Dalla-Salda et al., 2011). Other studies focused on growth performance instead of hydraulic performance and used ring width changes as response variable in order to avoid destructive measurements (e.g. Battipaglia et al., 2009; Eilmann and Rigling, 2012; Orwig and Abrams, 1997; Pasho et al., 2011).

The understanding of drought sensitivity of individual trees, provenances, tree species and entire forest ecosystems is an issue of utmost importance, as some ecosystems worldwide have experienced and more are expected to undergo significant increases in the frequency, duration and severity of drought periods due to climate change (Allen et al., 2010; Beniston et al., 2007). In forest ecosystems, drought periods were found to result in reductions of the gross primary productivity and led to carbon emissions (Ciais et al., 2005) as well as to increased tree mortality either due to direct die-offs or indirectly via boosted insect outbreaks (Allen et al., 2010). Consequently, among different silvicultural adaptation methods, the planting of different provenances of the present species or even the planting of alternative tree species has been suggested as effective management option (e.g. Berry et al., 2002; Bradley St Claire and Howe, 2007; Hamann and Wang, 2006; Jandlet al., 2015; Matyas, 1996; Rehfeldt et al., 1999).

The genus *Abies* encompasses, amongst others, ten species that are distributed around the Mediterranean, of which only one species (silver fir, *Abies alba* Mill.) is also part of temperate and alpine forests. In the low mountain ranges of western, southern, south-eastern and Central Europe, silver fir is a main component of the forest climax vegetation. Recent comparisons of tree species suggest that silver fir is more resilient to climate change (and associated phenomena like bark beetle outbreaks and storm damage) than other conifers of temperate forests (Zang et al., 2011). Currently economically less important Mediterranean firs have small, partially disconnected distribution ranges, but they could substitute *A. alba* or other conifers in temperate European forests if increasing temperatures and decreasing precipitation endanger present tree species compositions. However, a systematic analysis of drought sensitivity within the genus *Abies* and among populations of silver fir across several drought periods is not available so far.

In our study, data was taken from a long-term provenance trial comprising 10 provenances of *A. alba* and 5 Mediterranean *Abies* species. This trial site was established in 1970 with the explicit objective to investigate the drought reaction of *Abies spec* (Mayer et al., 1980) and located in eastern Austria, where severe summer droughts occur frequently (see for example Büntgen et al., 2010). In our analysis, we aim at the following questions: (i) Do the various species and provenances of the genus *Abies* respond differently to drought situations? (ii) Can wood properties (e.g. ring width, ring density) be used to predict the reaction of species or provenances to drought events? (iii) Does the climate at the origin of seed of a respective species or provenance explain its specific reaction?

## 2 Materials and methods

### 2.1 Trial site and plant material

**Fig. 1:**
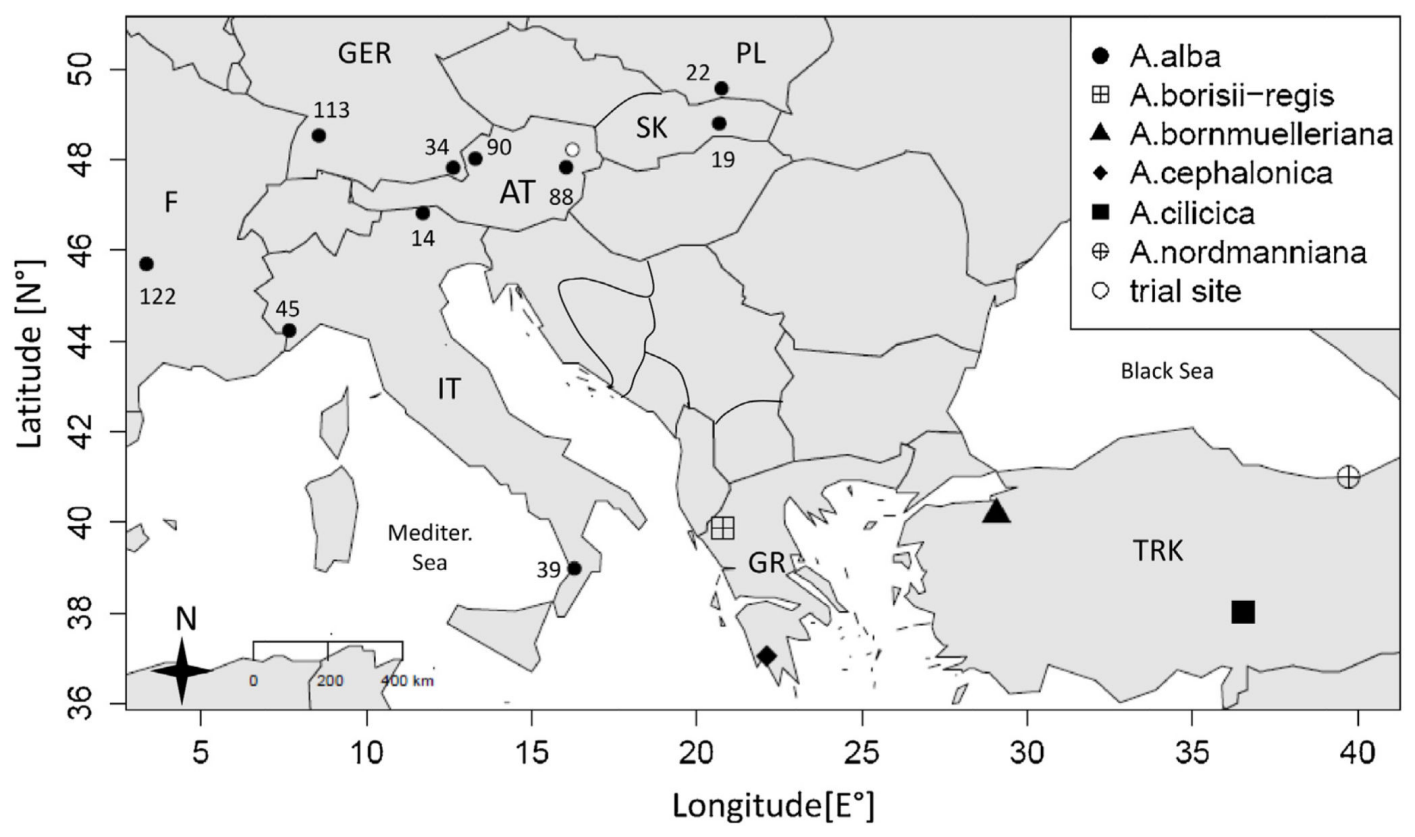
Overview of seed origin of the analyzed species and provenances of the genus *Abies*. Country abbreviations are: AT (Austria), F (France), GER (Germany), GR (Greece), IT (Italy), PL (Poland), SK (Slovakia), TRK (Turkey).

The trial site is located in eastern Austria at the border of the sub-pannonian Vienna basin. It is placed on a moderate south-west slope at 290 m a.s.l. Mean annual air temperature is 8.6 °C and the annual precipitation sum is 650 mm with 270 mm during the vegetation period (ZAMG weather station “Hohe Warte” 1970–2010). Seed material of the Mediterranean fir species originated from Turkey and Greece and the provenances of silver fir from across its natural distribution area (Fig. 1). The trial was planted in 1970 as a randomized block-design with plant spacing of 0.5 × 1.0 m using two- and three-year-old seedlings (Mayer et al., 1980). In March 2012, all specimen, for which at least eight trees were available were sampled by taking two cores per tree at breast height (Table 1). This included ten provenances of *Abies alba*, four Mediterranean fir species (*A. bornmülleriana, A. cephalonica, A. cilicica* and *A. nordmanniana*), and the natural hybrid of *A. alba* and *A. cephalonica: A*. x *borisii- regis* (Mattfeld, 1926; Bella et al., 2014).

### 2.2 Core sample preparation, X-ray densitometry and removal of age-trend

Approximately 1.4 mm thick cross sections of each core, produced with a double-blade circular saw, were placed on microfilms and exposed to a 10 kV (24 mA) X-ray source for 25 min. Microfilms were analyzed using WinDENDRO 2009 (Regent Instrument, Quebec, CAN). This procedure provided measurements for mean ring density (RD), early-wood density (ED), late-wood density (LD), minimum density (MIND) and maximum density (MAXD) for each year as well as for ring width (RW), earlywood width (EW), latewood width (LW) and latewood proportion (LWP). Density parameters were measured in kg/m^3^ while ring width parameters were measured to the nearest 0.001 mm. LWP expresses the relative proportion of latewood compared to total ring width and is therefore given in percentages. Values from the two cores of the same tree were averaged to reduce non-climatic effects (such as reaction wood and faults in the wood tissue) and to account for potentially missing data of the youngest and oldest tree rings. For further analysis of identification of drought years (see below), we removed the biologically caused age trend occurring in the short time series of ring width by using a flexible 15-year cubic smoothing spline that was fitted and visually evaluated using the dplr package of the software R (Bunn, 2010; R Development Core Team, 2008).

### 2.3 Identification of drought events and evaluation of performance under drought

To identify drought periods with subsequent effects on tree growth and wood density, we calculated the standardized precipitation index - SPI according to McKee et al. (1993). The SPI is based on monthly precipitation time series (in our case from 1970–2010) and relates the actual precipitation deficit to the mean and standard deviation of the time series. In contrast to drought indicators with fixed time scales such as the PDSI (Guttman, 1997; Vicente-Serrano et al., 2010), the SPI is able to identify and differentiate between frequently occurring short- and longer drought events, because the duration of the tested drought/precipitation period can be modified from few months up to years. We chose two different time scales (1- and 3-months, respectively) since these time spans represent biological meaningful periods of water-shortage for trees and are likely to occur in central Europe. For calculation of SPI we used the program *SPI SL 6* (NDMC, 2014). Years were considered as drought years, when they showed a severe or extreme shortage in water supply (severe drought: SPI ≤ −1.50; extreme drought: SPI ≤ −2.00) within the vegetation period (April to August). To assess a trees performance during these drought events, we calculated four indices of drought reaction following Lloret et al. (2011): resistance (Res), recovery (Rec), resilience (Rsl), as well as relative resilience (rRsl). Resistance can be characterized as a trees’ ability to withstand a period of low water supply without showing a perceptible drop in ring width (i.e. trees with a Res = 1 do not show any decrease in annual increment) and is calculated by the ratio between ring width during (Dr) and before a drought event (preDr). Recovery describes the ability to restore from increment drops experienced during a drought (here, Rec = 1 stands for persistence at low growth levels after a drought, whereas Rec>1 indicates an immediate increase) and is given by the ratio of ring width after (postDr) and during (Dr) a drought event. Resilience is the capacity of a tree to reach predrought levels of ring width after a drought event (Rsl = 1 means full restoration, Rsl < 1 indicates further decline in growth). It is calculated by the ratio of ring width after (postDr) and before (preDr) a drought event. The relative resilience (rRsl) indicates, if the effect of a drought is still persisting (rRsl < 1) after disturbance and gives information of how fast a tree is able to recover to a pre-drought level (higher values indicate faster recovery), by taking the magnitude of growth reduction during the drought into account. rRsl is given by ((postDr-Dr)/preDr). Pre-drought and post-drought ring widths were calculated as average values for a three-year period before or after a year with drought. All four indices were derived from the raw, untransformed ring width series of each tree, because the biological age-related growth decrease can be neglected within the relatively short time frame of each individual drought event (Pretzsch et al., 2013).

### 2.4 Statistical analysis

Firstly, we tested if the observed variation of wood properties (calculated as average values across the complete core age) and drought reaction indices of the dataset is based on differences between *Abies* species or *Abies alba* provenances using the variance components analysis (procedure MIVQUE: Minimum Variance Quadratic Unbiased Estimation, *Statistica 9.0*, StatSoft inc., 2013), where both species and provenances were treated as random variables within one analysis. This variance components analysis is able to account for the unbalanced contribution of species and provenances to our dataset and should help to justify further analysis steps. Thereafter, intra-specific variation was investigated only across *A. alba* provenances, while analysis of inter-specific variation included all sampled *Abies* species. Because *A. alba* was highly overrepresented in the dataset for inter-specific comparison (see Table 1), we chose a random subset of *A. alba* individuals across all provenances equal to samples sizes of the other fir species in order to achieve a ‘fair’ comparison. Inter- and intra-specific differences for wood properties as well as for drought reaction indices were calculated by ANOVA using ‘provenance’ and ‘species’ respectively as categorical fixed variables (procedure *lm* in R). Pairwise differences between species/provenances were assessed with Tukey’s *posthoc* test using the package *multcomp* vers. 1.3–8 in R (R Development Core Team, 2008).

In previous studies, wood properties were found to be correlated to drought sensitivity of species and provenances (e.g. Rosner et al., 2014; Martinez-Meier et al., 2008a,b) and thus were suggested to be suitable traits for the selection of drought resistant trees. To test if this relationship also holds for *Abies* spec., we calculated Pearson’s correlation coefficient between the wood properties (RW, RD, etc.) averaged across the complete core age and the drought reaction indices of the identified drought events.

The specific drought reaction of species or provenances may be a result of adaptation to the climate conditions of a species distribution and local habitat. Therefore, we tested if the observed differences in drought reaction and wood properties are correlated to the climatic conditions and geographic coordinates of the seed origin. We used 19 bioclimatic variables from the World-Clim database (Hijmans et al., 2005) from which 11 variables refer to temperature and 8 to precipitation at the place of seed origin (Table 2).

## 3 Results

### 3.1 Identification of drought events

**Fig. 2:**
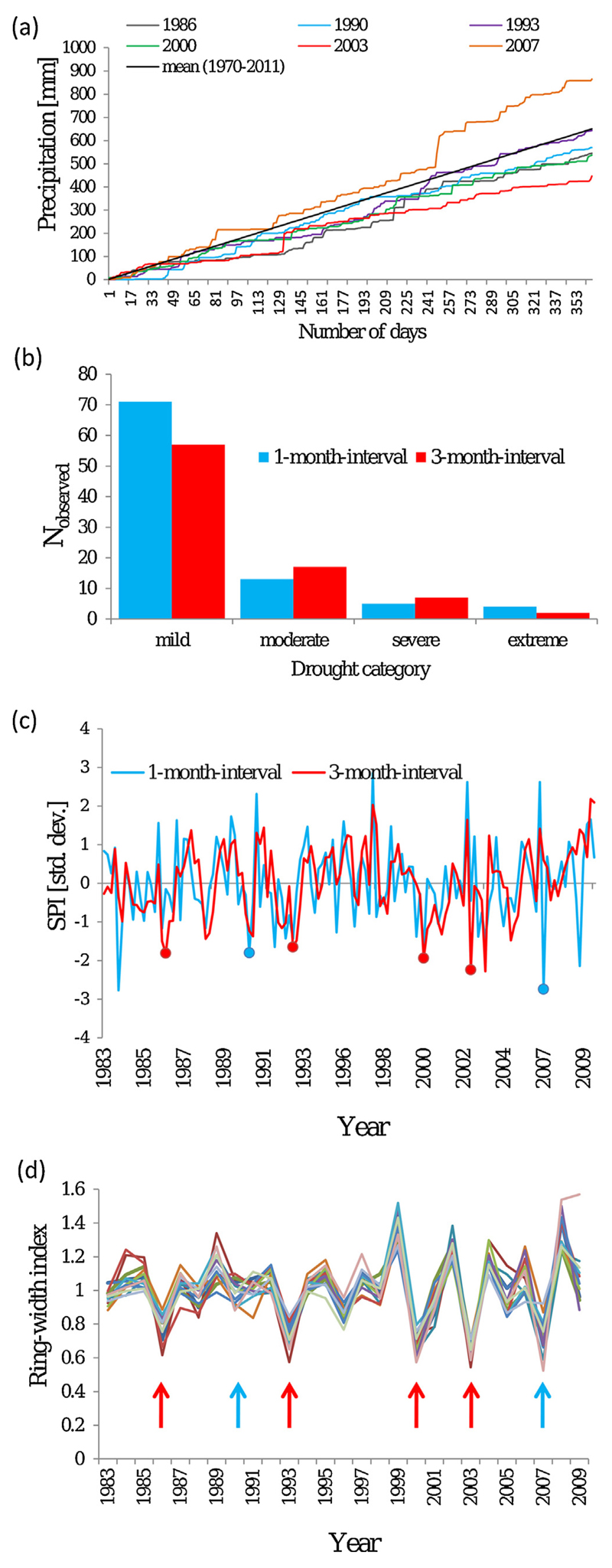
(a) Cumulative precipitation sum of the analyzed drought years; (b) Frequency of droughts in each category (classification of McKee et al., 1993) from 1970 to 2011 on two different time scales (blue and red); (c) Standardized precipitation index (given in standard deviations) across the time series of 1983–2009 on two different time scales (blue and red), points indicate either severe or extreme dry years that were considered for the analysis; (d) Ring width indices averaged across species and provenances, respectively. Species and provenances are shown in different colors to illustrate differences in drought reaction. Ring width indices are given dimensionless, arrows are indicating event years in coincidence with low SPI values on two different time scales (blue and red).

Across the years 1983–2010 we found six years with SPI values indicating either a severe (1990, 1993, 2000) or extreme (1986, 2003, 2007) precipitation deficit. Furthermore, two drought periods appeared at the beginning (1983) and the end (2009) of the time series. The latter two droughts could not be included in the analysis, because for the first and the last years, the data of many individuals were missing and thus calculations of pre- and post-drought periods were not possible. In 1986, 1993, 2000, 2003 and 2007, observed droughts were in coincidence with remarkable growth reductions of trees (Fig. 2b and c). In contrast, tree growth was only slightly affected by the drought in 1990 and some provenances even showed ring width indices above average growth in this year. The drought 2007 can be characterized as a severe spring drought (22 days without any precipitation in series followed by another 14 day series with only 2 mm rainfall in between from end of March until beginning of May, Fig. 2a), although the total precipitation sum of this year was above the long-term mean. Since the drought 2000 was closely followed by the drought 2003, a two-year average period was used as reference period for preDr_00_, postDr_00_, preDr_03_ and postDr_03_ to avoid overlapping of the reference periods with a drought event.

### 3.2 Inter- vs. intra-specific variation

**Fig. 3:**
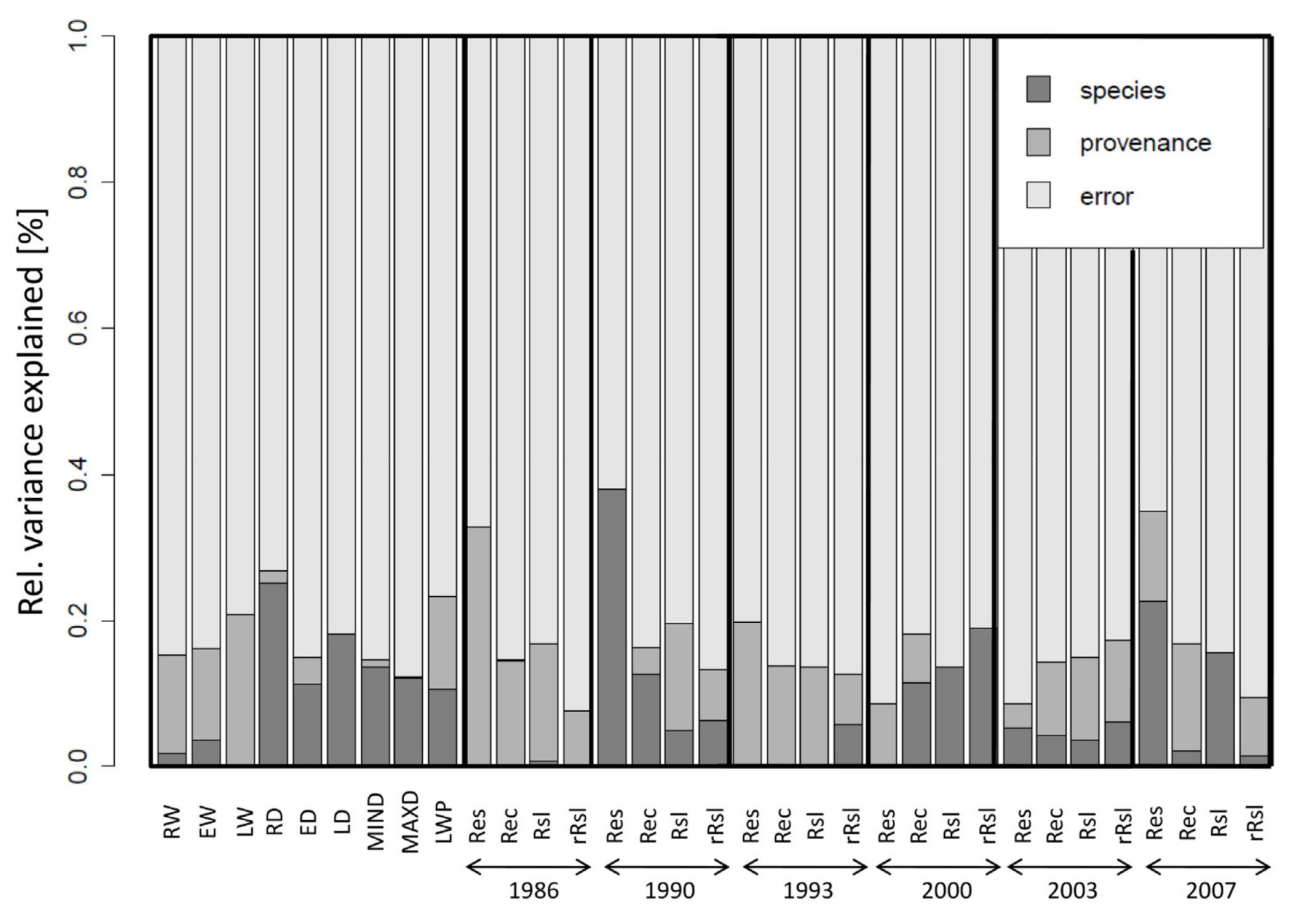
Relative variance in wood properties and drought response measures explained by species and provenance, respectively, using Minimum Variance Quadratic Unbiased Estimation (M1VQUE0).

Comparing the contributions of species and provenances to the observed variation of wood traits and drought response measures revealed that *Abies* species explained 10–20% of variance of the wood density parameters (RD, ED, LD, MIND and MAXD), but only a negligible amount of the variance of ring width measure (RW, EW, LW, LWP). In contrast, *A. alba* provenances are responsible for 10–15% of variation among ring-width measures but explain only a non-significant proportion of the variance in ring density measures (Fig. 3). Moreover, species and provenances contributed differently to the variance of drought response indices: while inter-specific variation was found to explain variance of recovery, resilience and relative resilience in 2000 as well as of resistance and resilience 2007, intra-specific variation contributes mainly to the variance of all four drought reaction indices in 1986 and 1993 as well as for resistance in 2000 and recovery and relative resilience in 2007. Due to these contrasting contributions of species and provenances to the analyzed wood traits and drought response indices, all further analysis has been made separately for *Abies* species and *A. alba* provenances.

Analysis of variance confirmed the variance component analysis: among species, significant differences were found for latewood and maximum density (Table 3), whereas among *A. alba* provenances, significant differences were found for ring width, earlywood width, latewood width and latewood proportion (Tables S4 and S5 Suppl.). For drought reaction indices, significant differences among species were found for all measures in 2003 and 2007 (except Rsl_07_), as well as for Rec and rRsl in 1993 and 2000. Among provenances, significant differences were only found for Res, Rec and Rsl in 1986, for Rec, Rsl and rRsl in 1990, for Res and Rec in 1993 and for Res in 2000.

**Fig. 4:**
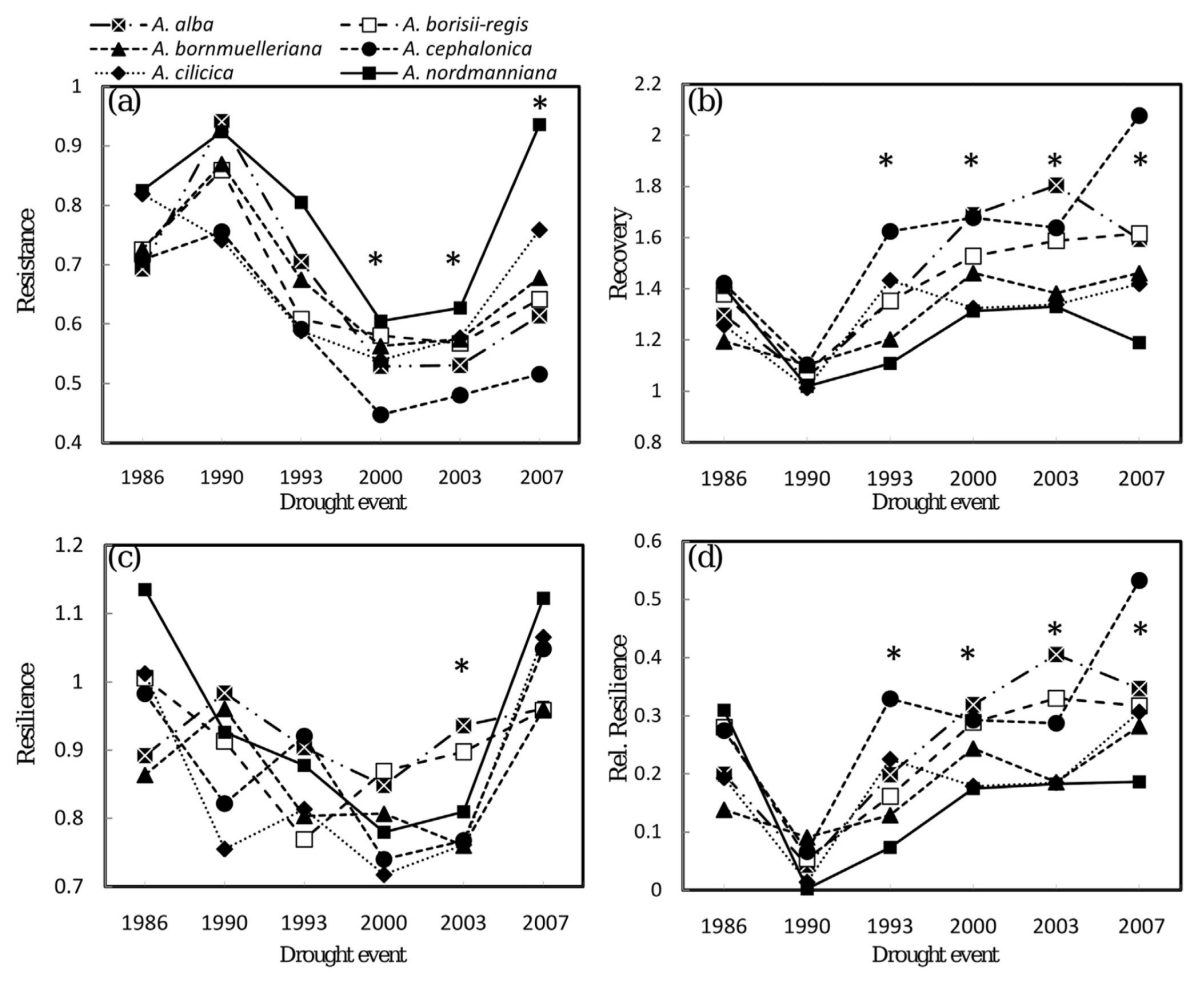
Plasticity of drought reaction among species for the four different drought response measures. Drought response measures on the *y*-axis are given as dimensionless numbers and the average value for each species is shown. Asterisks indicate that significant pairwise differences were observed during this drought event. Lines are only drawn for illustration and to show if stability or instability in drought reaction across several drought events was observed.

**Fig. 5:**
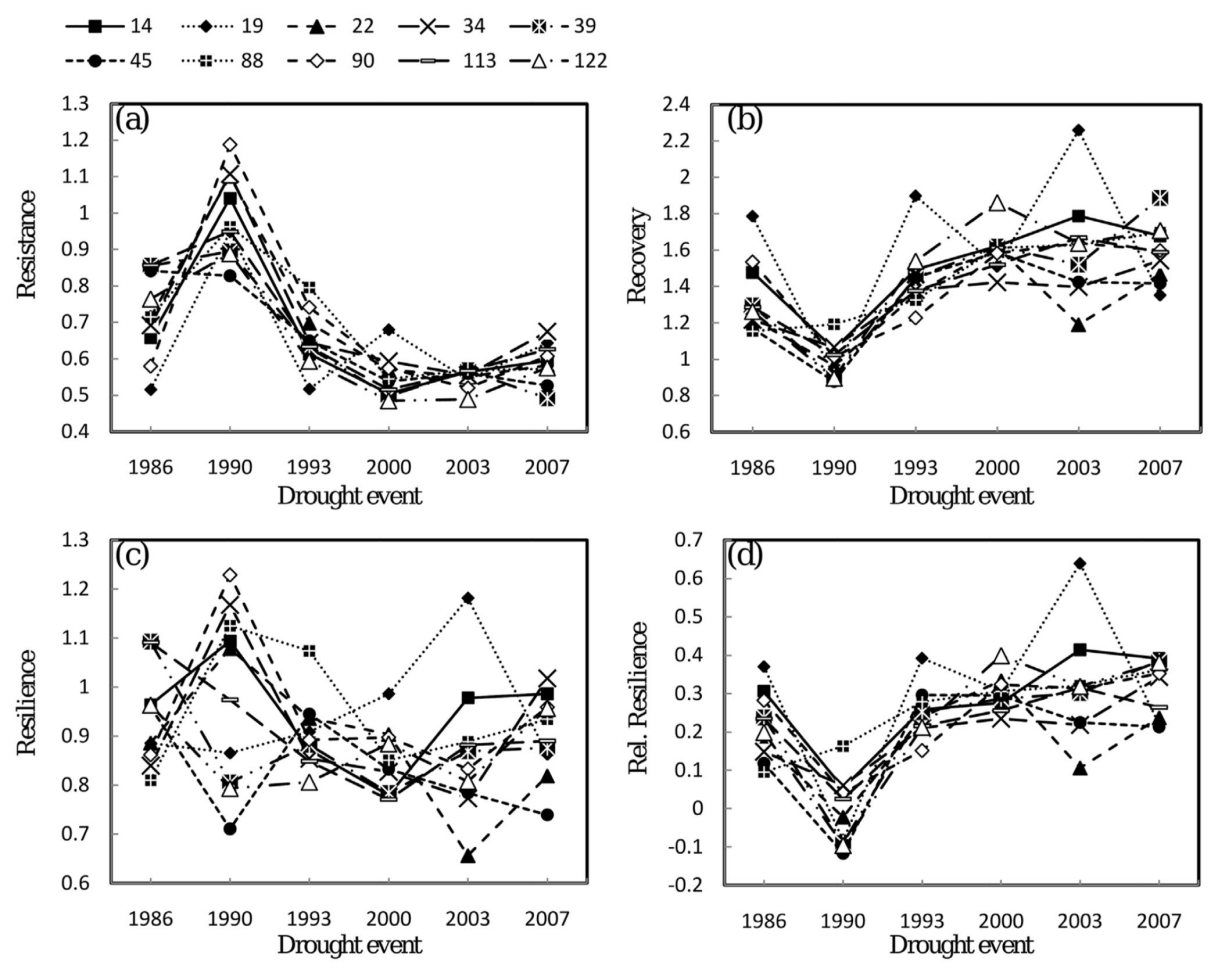
Plasticity of drought reaction among provenances of *A. alba* for the four different drought response measures. Drought response measures on the *y*-axis are given as dimensionless numbers and the average value for each provenance is shown. Lines are only drawn for illustration and to show if stability or instability in drought reaction across several drought events was observed.

Pairwise posthoc comparisons among species and provenances, respectively, indicate that the differences in drought reactions follow a consistent pattern across species, but not among provenances. Among the different *Abies* species, *A. nordmanniana* showed the highest resistance across five of the six drought periods. In 2003, the observed differences were significant between *A. nordmanniana* and *A. cephalonica*, and in 2007, *A. nordmanniana* and *A. cilicica* showed significant higher resistance than all other *Abies* species (Figs. 4 and 5 and Suppl. Table S1). In contrast, A. *cephalonica* was found to have the highest recovery in 1986, 1990, 1993 and 2007, while *A. nordmanniana* had the lowest in four of six analyzed drought periods. *A. alba*, as the most widespread of all analyzed species, showed intermediate ranks for drought reaction indices with the exception of the year 2003, where it performed best in recovery, resilience and relative resilience. *A. x borisii-regis*, the natural hybrid of *A. alba* and *A. cephalonica*, did not reveal significant different drought performance compared to its parent species for any of the four response indices (Suppl. Table S1).

Pairwise comparisons of the drought response measures among provenances of silver fir also revealed significant differences, but no consistent pattern of ‘best-performing’ or ‘worst-performing’ provenances across all analyzed drought periods could be found (ranks for some *A. alba* provenances even changed from 1 to 10 between certain drought events). For example, in 1986, provenance 39 (Calabria) showed the second highest rank in resistance and provenance 19 (Stara-Voda) the lowest, whereas in 2000 provenance 39 had the second lowest rank in average resistance and provenance 19 the highest. A comparison of the average ranks of resistance across all six drought events places provenance 34 (Siegsdorf) in first and provenance 122 (Auvergne) in last place. Focusing on recovery, provenance 122 had the second highest average rank, whereas provenance 34 had only the third lowest average rank (Suppl. Tables S2 and S3).

### 3.3 Wood traits as indicator of drought sensitivity

Identifying wood characteristics related to drought response that can be screened non-destructive even before drought events occur would be a valuable tool for breeding programs and adaptive forest management. When we tested for correlations between ring width parameters and drought response indices, positive correlations were obtained to Rec, Rsl, rRsl in both datasets (provenances and species), but higher and more significant correlations were found to the intra-specific data. The correlations between wood density parameters and drought response measures were more complex. Among species, density was negatively correlated with resistance of all drought events (significant correlations between RD and Res_00_ as well as between ED and Res_86_) and positively correlated with recovery and relative resilience (Fig. 5). Among provenances variation of density parameters showed much less relations to drought indices: here, the latewood percentage (LWP) was negatively correlated with Rec_86_, Rsl_86_, rRsl_86_ and rRsl_03_. Minimum density (MIND) showed significant negative correlations to Rec, Rsl and rRsl in 2007. Only two significant positive correlations for intra-specific data were found: between maximum density (MAXD) respectively LD and Res_03_.

### 3.4 Climate predictors of wood trait and drought reaction

**Fig. 6:**
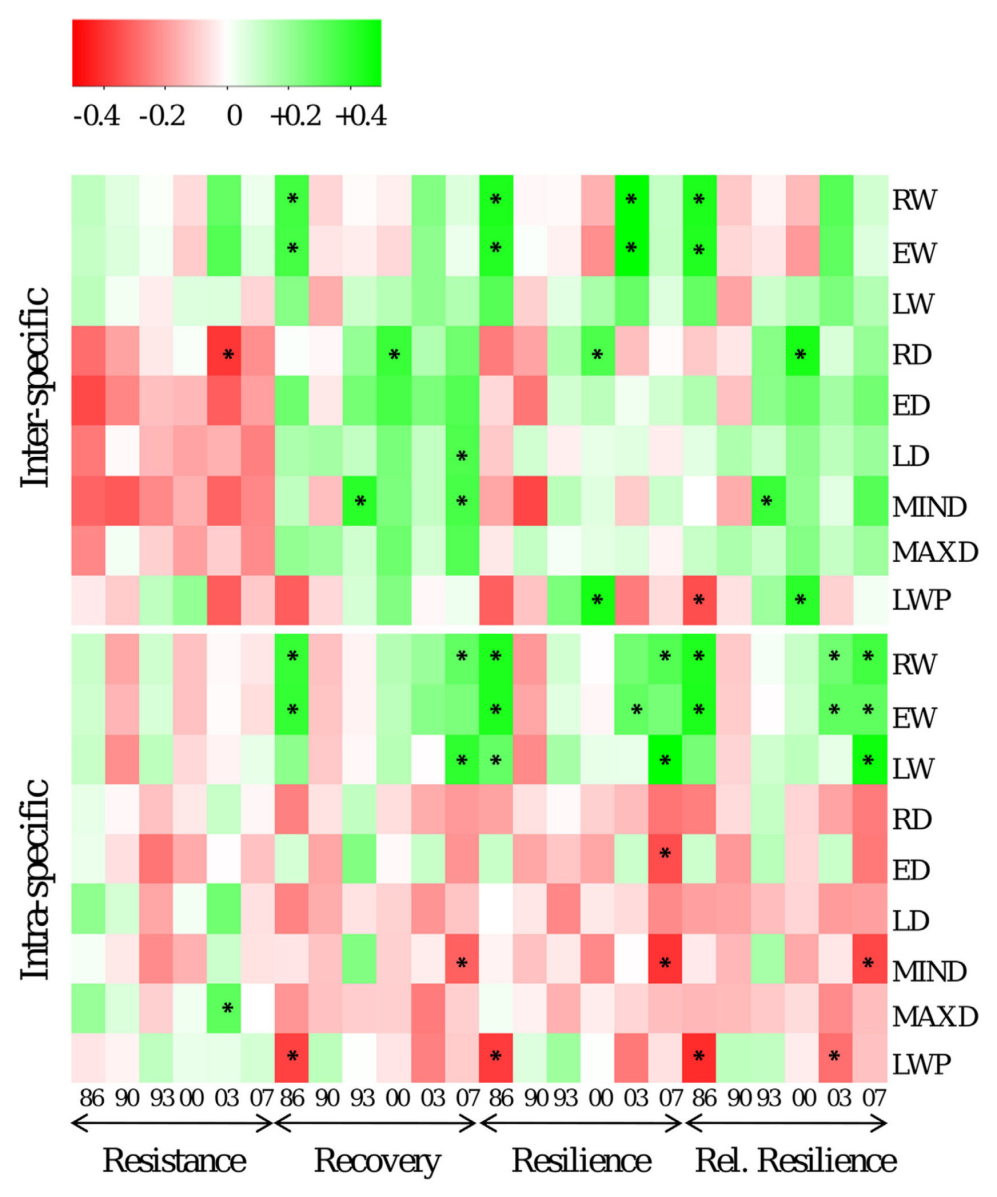
Heatmap showing the correlation between wood properties (right) and drought response measures (bottom) on inter-specific (upper matrix) and intra-specific (lower matrix) level. Asterisks indicate that correlation is significant on *p* < 0.01. Note that these heatmap shows broad trends, since *p*-values are not corrected for multiple comparisons.

Inter- and intra-specific differences were also found for correlation patterns between drought response measures and geographic/bioclimatic variables. For *Abies* species, the strongest correlations were obtained between the longitude and various drought response indices: here species from the eastern Mediterranean showed higher resistance, but lower recovery, resilience and relative resilience (Fig. 6). From the 19 bioclimatic variables tested, six precipitation variables (AP, PwM, PdM, PS, PwQ and PCQ) showed positive correlations with Rec, Rsl and rRsl throughout the *Abies* species. Significance of these correlations at *p* < 0.01 was obtained for the drought events 2000 and 2007. For the same dataset, correlations with temperature variables were rather weak and significant only for isothermality (ISO). Correlations of the intra-specific variation to bioclimatic data revealed significant correlations only to Res_86_ and Res_07_ as well as to Rsl_86_.

**Fig. 7:**
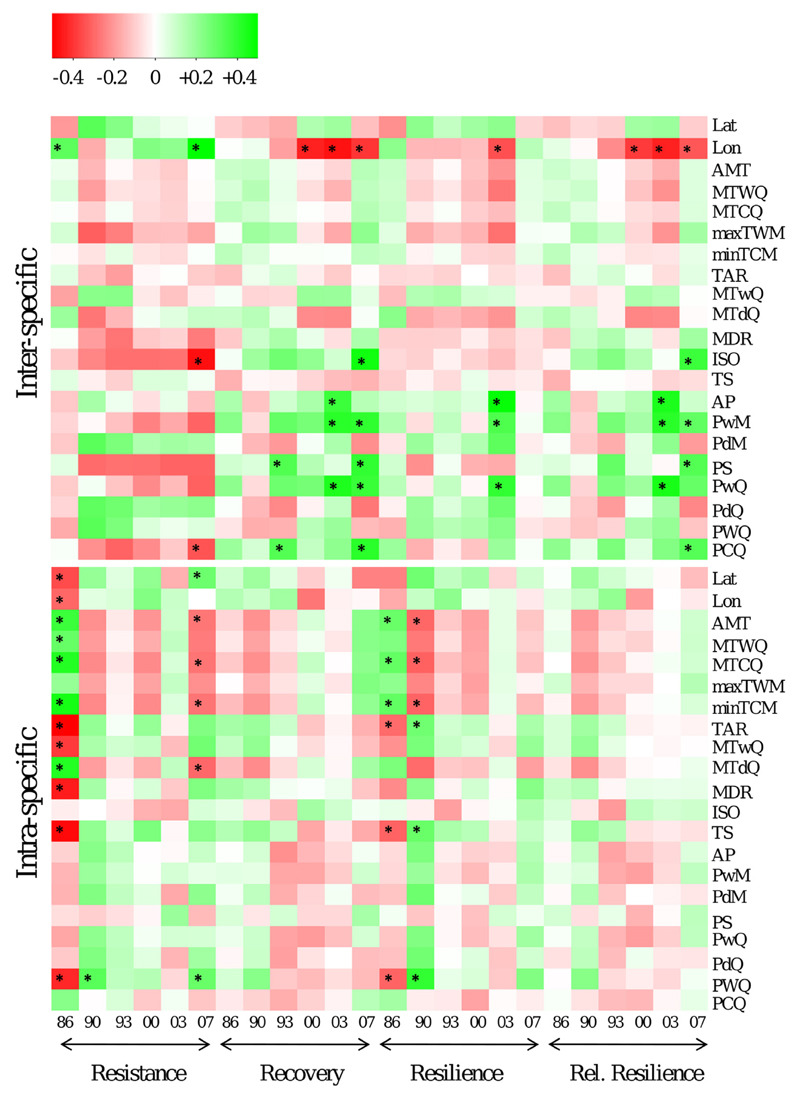
Heatmap showing the correlation between bioclimatic variables (right) and drought response measures (bottom) on inter-specific (upper matrix) and intra-specific (lower matrix) level. Asterisks indicate that correlation is significant on *p* < 0.01. Note that these heatmap shows broad trends, since *p*-values are not corrected for multiple comparisons.

**Fig. 8:**
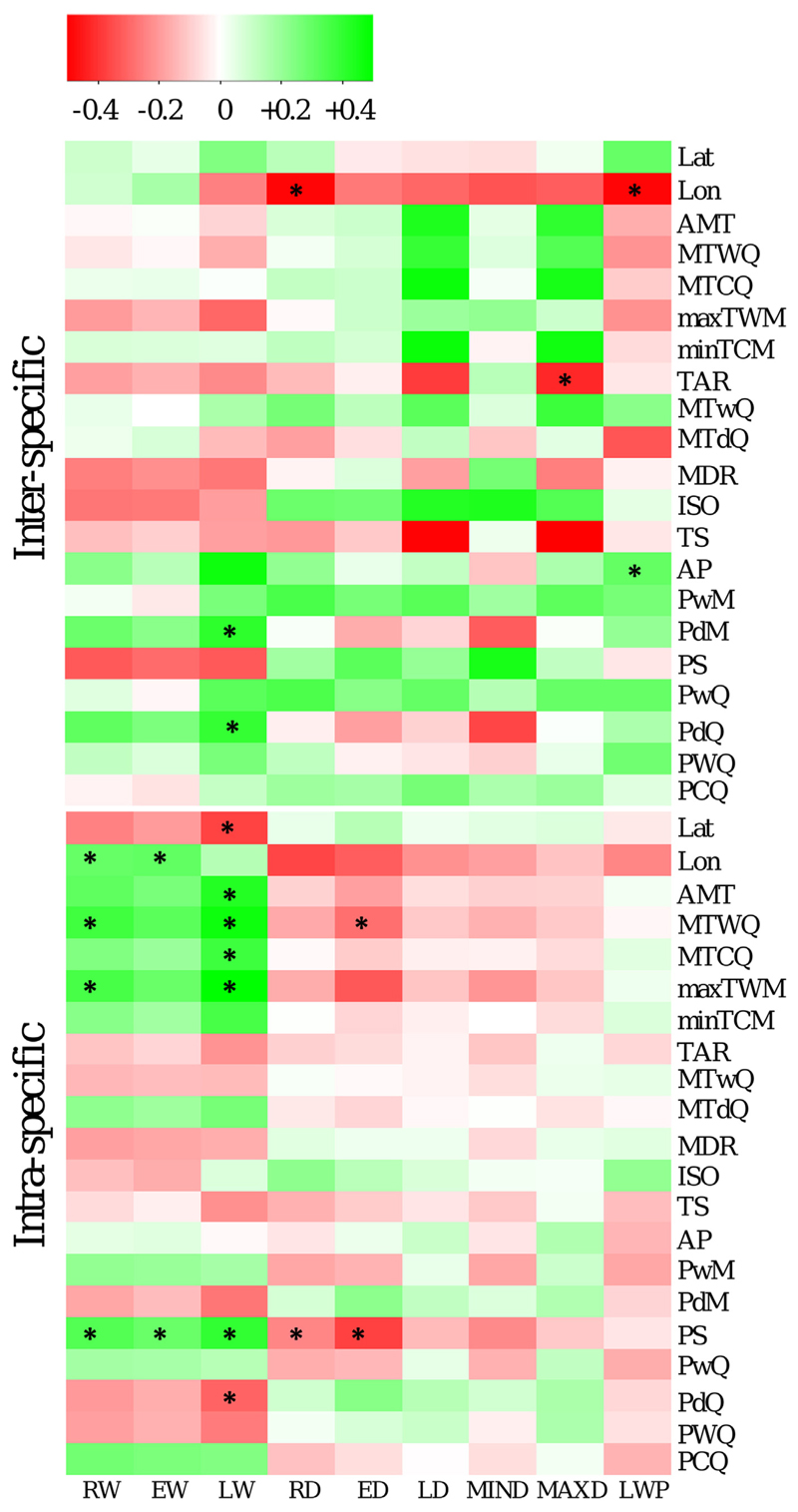
Heatmap showing the correlation between bioclimatic variables (right) and wood properties (bottom) on inter-specific (upper matrix) and intra-specific(lowermatrix) level. Asterisks indicate that correlation is significant on *p* < 0.01. Note that these heatmap shows broad trends, since *p*-values are not corrected for multiple comparisons.

Wood properties showed only few significant correlations to bioclimatic and geographic variables for both datasets, whereas both inter- and intra-specific ring density variation was negatively correlated to longitude (*p* < 0.01), suggesting that provenances and species from more eastern origin have lower wood density (Fig. 7). This is accompanied by a tendency for higher ring widths toward eastern seed origin with significant correlations for *A. alba* provenances. Very few bioclimatic variables were correlated to wood properties, but most notably the seasonality of precipitation (positively correlated with RW, EW, LW and negatively with RD and ED in the intra-specific dataset). A positive correlation (*p* < 0.01) was found both for RW and LW with mean temperature of the warmest quarter (MTWQ) and mean temperature of the wettest quarter (MTwQ) on intra-specific level. Significant correlations between bioclimatic variables and wood properties were generally less abundant for inter-specific data. Correlations between wood properties and seasonality of precipitation (PS) showed a complete reverse pattern compared to intra-specific data (by trend negative for RW, EW, LW and positive for all density parameters) (Fig. 8).

## 4 Discussion

### 4.1 Drought years

Five severe or extreme drought periods with significant effects on tree growth occurred at the trial site in eastern Austria during the life span of the investigated trees. The drought events in 1986, 1993 and 2003 were characterized by the SPI (McKee et al., 1993) as droughts of long duration but lower intensity on an intermediate time-scale (three month), whereas the drought in early spring 2007 can be characterized as an event with high intensity and relatively short duration. Nevertheless, the negative consequences of this event for tree growth were of a similar order of magnitude as the drought in 2003, this being considered to be the most significant drought event in Central Europe and used as a reference for investigating drought effects on forest ecosystems and other biological systems (e.g. Ciais et al., 2005; Dalla-Salda et al., 2009; Levesque et al., 2014; Pichler and Oberhuber, 2007; Taeger et al., 2013). Despite the fact, that short-term drought events are rarely discussed in the current literature so far, our study strongly suggests that these drought events need similar attention in climate change studies. The drought in 1990 had no significant effect on tree growth despite its severe character and a likely explanation is that tree-ring formation was almost completed when this drought occurred. Indeed, monthly SPI data indicate, that this drought had occurred late in the growing season in August while the other five had occurred during April and June (see Fig. 2a).

### 4.2 Variation in drought response within and among Abies species

We found species-specific and provenance-specific differences in wood properties and drought response measures during six drought events. The drought reaction of *A. alba* provenances differed significantly during the three severe drought events 1986, 1990 and 1993, while *Abies* species showed significant differences during the extreme events 2003 and 2007. This contrasting behavior could be caused by the severity and/or the seasonal occurrence of the drought events: in 1986, 1990 and 1993 drought might have been too weak to provoke a different reaction of the various species, but strong enough to reveal intra-specific differences among provenances. In contrast, the stronger drought events in 2003 and 2007 might have exceeded a certain threshold above which all *A. alba* provenances were equally affected, but caused contrasting reactions among the different fir species. The underlying genetic basis of the contrasting drought reaction is probably the inherent genetic variation of the species. Although *A. alba* provenances originate from a wide geographical range and from different phylogeographic lineages, they are phylogenetically much more similar and connected via contemporary gene flow (Liepelt et al., 2002) than the sister *Abies* species (Liepelt et al., 2010). Thus, differences in adaptive traits related to drought reaction are more pronounced among rather than within species. This explanation is supported by the ranking pattern of drought response indices across the six drought events, which is consistent among *Abies* species but not among *A. alba* provenances. *Abies* species tended to keep their ranks for a specific drought response index across the different events while *A. alba* provenances often changed their ranks even between two consecutive drought events.

Besides specific morphological or physiological drought adaptations within species or provenances, genetic correlations between the estimated drought response measures and other quantitative traits could be responsible for the observed drought sensitivity, too. For the present *Abies* trial, surveys of bud burst are available from the assessment on the young seedlings in 1971 (Kral, 1980), showing that bud burst in *A. nordmanniana* took place significantly later (e.g. in 1971 on May 1st) than for all other species and provenances (e.g. on April 18th for *A. cephalonica*). Also Aussenac (2002) classified *A. nordmanniana* as a late flushing species. These differences in time of flushing can partly explain the high resistance of *A. nordmanniana* during the spring drought in 2007: while the early flushing species required high amounts of water to enfold the new shoots, the late flushing *A. nordmanniana* simply avoided drought stress because the metabolic processes during spring were still down-regulated. In forest trees, the time of bud burst is highly stable within individuals and shows high heritability (Beuker, 1994; Gailing et al., 2009; Schueler and Liesebach, 2015; Scotti-Saintagne et al., 2004), suggesting that genetic variation and plasticity of tree phenology need to be considered when selecting for drought adapted phenotypes.

Generally, the quantitative and adaptive genetic variation of silver fir and the variation among *Abies* species have been investigated only in very few studies. Analyzing adaptive and non-adaptive traits in southwestern populations of silver fir in France on four-year-old seedlings, Sagnard et al. (2002) observed high trait variation within but low trait variation among provenances. For drought-resistance traits, the variation among provenances ranged only from 6.6 to 6.8%, while for growth traits, variation among populations accounted for 10 to 17.9% (Sagnard et al., 2002). This is very similar to our study, where significant differences among provenances were found for ring width measures, but only for few drought response measures. The high variation among provenances in growth performance has also been demonstrated by a wide number of provenance experiments with silver fir (Hansen and Larsen, 2004; Mayer et al., 1980). In contrast to frost hardiness and cold adaptation (Howe et al., 2003; Savolainen et al., 2004), the genetic variation of drought sensitivity and its application in breeding programs for *Abies spec*., and generally for other conifers, has rarely been investigated. Only recently, the drought response of different tree species (e.g. Eilmann and Rigling, 2012; Levesque et al., 2014; Zang et al., 2014) or provenances within species (Dalla-Salda et al., 2009; Eilmann et al., 2013; Martinez-Meier et al., 2008a,b) has become an important research objective as part of adaptive forest management in climate change (e.g. Jandl et al., 2015).

A final rating of drought sensitivity among and within species should consider all aspects of drought response, i.e. all four drought response measures, because species/provenances with high resistance might not necessarily show good recovery. Within the present paper, resistance strictly refers to the drought response measures Res as defined by Lloret et al. (2011) and should not be confounded with a general ability to avoid any drought symptoms. Species, which revealed high resistance (i.e. low reductions in annual increment) like *A. nordmanniana* for Res_1986–2007_ also showed the lowest recovery and relative resilience, while those with low resistance showed higher recovery (see average ranks for Res, Rec and rRsl in Table S1). These differences can be interpreted as different life-strategies or long-term adaptations: *A. nordmanniana*, originating from the edge of the Caucasian mountains, a region with relatively high precipitation and moderate temperature during the growing season (Table 1), kept on growing during drought, while *A. cephalonica*, originating from a region with only 4 mm precipitation in the driest month, reduced its growth quickly. After a drought event, the resource budget of the high-resistance species might be depleted due to the dissimilation of stored carbohydrates during drought resulting in lower radial increment in the following years (Adams et al., 2009). This trade-off is also supported by the correlations between the climate conditions of the seed origin of species and provenances and the drought response measures. On inter-specific level we found the precipitation in the wettest month and in the wettest quarter, which are both decreasing by trend with longitude (Suppl. Fig. S1), to be best predictors of drought response, confirming the findings that species from the eastern Mediterranean basin showed higher resistance, but lower recovery and lower relative resilience (Fig. 6). At the Mediterranean, the wettest month (wettest quarter) is outside the growing season in winter (either from Oct-Dec or from January to March, data not shown). Thus, our results are in accordance with findings from Williams et al. (2013), who have underpinned the importance of cold-season precipitation as a driving force for mitigating forest drought-stress in the southwestern United States. Trade-offs between growth reductions (=resistance) and growth recovery after drought events have also been discussed within (Galiano et al., 2011) and among other tree species (Pretzsch et al., 2013; Zang et al., 2012). As our data do not include any estimate of tree mortality, neither our present study nor former investigations could relate the resistance-recovery trade-off to the real fitness of individual trees or populations. Reports from the drought-prone southwest North America provide contradictory arguments for the relation between drought response and mortality. While McDowell et al. (2010) found that ponderosa pine (*Pinus ponderosa*) trees more sensitive to climate events showed higher mortality, various other studies suggested that trees with strong growth response in relation to drought are more likely to survive the drought event (e.g. Macalady and Bugmann, 2014; Ogle et al., 2000). It is speculative without long-term observations and more detailed physiological measures, if one of these relationships also holds for the observed variation among populations and species in case of European fir species. Nevertheless, if the predicted drought scenarios became reality, then the results could provide rough guidelines for future forest management. Firstly, species for future reforestation schemes need to be related to the respective forest site. In particular, the expected seasonal occurrence of drought would define which species were planted. Secondly, the proposed fir species might be selected in dependence of the expected primary forest function. If stable wood production with regular annual increment and density is expected, *A. nordmanniana* might be a good choice. Other ecosystem services or ecosystem stability might rather benefit from other fir species. Although *A. alba*, the most widespread fir species in our study, did not show strong intra-specific variation in drought response, it shows a similar response as *A. cephalonica:* low resistance and fast recovery. An immediate substitution of silver fir in drought prone temperate forests of Central Europe with Mediterranean firs seems not to be required at present. However, given the small and scattered distribution of Mediterranean firs and the increasing climatic stress in their natural distribution, a managed translocation toward northern mountain ridges might be needed to safeguard endangered genetic diversity (Alizoti et al., 2011; Schueler et al., 2014). Drought resistance certainly is an important quantitative trait and should be considered in translocation schemes, but being aware that besides abiotic interactions also biotic interactions with the wider forest communities (Kranabetter et al., 2015) need to be considered in risk and benefit analysis (Iverson & McKenzie, 2013).

### 4.3 Wood characteristics as screening tool for drought sensitivity

Can genetic correlations between wood characteristics and drought response measures be used to develop screening tools in breeding programs or to identify drought-vulnerable individuals for forest management operations? Previous studies reported moderate to strong negative relationships between wood density and a trees’ vulnerability to cavitation on intra-specific level (e.g. Dalla-Salda et al., 2011; Rosner et al., 2014) and inter-specific level (Hacke et al., 2001), suggesting that wood density might be a valuable selection criterion. When we tested for similar correlations among silver fir provenances, we could not confirm this correlation for mean ring density, and only found few moderate negative correlations between the minimum density and recovery, resilience and rel. resilience for the drought in 2007. We presume that the missing relationship between density measures and drought reaction within the species is mainly due to the low genetic variation among provenances. On the inter-specific level, average ring density revealed a negative relationship to resistance in 2003 and positive relationships to recovery, resilience and relative resilience for the drought in 2007. Drought response measures of the remaining drought events were not or only weakly correlated to ring density. Overall, our correlation analysis suggests that average wood density measures are poor predictors of drought sensitivity in the genus *Abies* and more specific physiological and hydraulic parameters are required. Anatomical and physiological studies suggest that in case of water shortage an early closure of stomata results into an efficient reduction of water loss in *Abies spec*. (Cochard, 1992; Peguero-Pina et al., 2011). This strategy probably avoids hydraulic failure that is caused by destruction of the water transport system. Our results also highlight the need for a study comparing the effectiveness of destructive versus non-destructive methods for estimating drought sensitivity and for developing easy and cost-effective screening tools to identify potentially drought-resistant genotypes.

## Appendix A. Supplementary data

Supplementary data associated with this article can be found, in the online version, at http://dx.doi.org/10.1016/j.agrformet.2015.08.268.

Figure S1

Supplemental Tables

## Figures and Tables

**Table 1:** Analyzed species and provenances of the genus Abies.

Label	Species	Country	Origin	Lat (N°)	Lon (E°)	AMT [°C]	AP [mm]	maxTWM [°C]	PdM [mm]	N
14	*A*. *alba*	IT	Brixen-Vintl	46°49′	11°43′	7.9	827	24.1	34	16
19	*A*. *alba*	SK	Stara-Voda	48°48′	20°41′	6.9	676	22.8	31	9
22	*A*. *alba*	PL	Nawojowa	49°34′	20°45′	7.9	735	24.2	31	10
34	*A*. *alba*	GER	Siegsdorf (Oberbayern)	47°49′	12°38′	7.5	1108	22.8	57	15
39	*A*. *alba*	IT	Serra San Bruno (Catanzaro-Calabria)	38°59′	16°18′	15.8	947	28.2	17	10
45	*A*. *alba*	IT	Valle Pesio (Cuneo)	44°14′	07°40′	8.9	897	21.6	43	10
88	*A*. *alba*	AT	Hohe Wand	47°50′	16°03′	7.6	767	23.3	37	11
90	*A*. *alba*	AT	Schneegattern	48°01′	13°18′	7.9	1257	23.3	75	9
113	*A*. *alba*	GER	Pfalzgrafenweiler (Schwarzwald)	48°32′	08°34′	8.2	949	22.5	64	16
122	*A*. *alba*	F	Maubert (Cantal-Auvergne)	45°42′	03°21′	10.4	649	25	32	14
73	*A*. *cilicica*	TRK	Göksun	38°01′	36°31′	9.2	599	29	7	10
75	*A*. *bornmuelleriana*	TRK	Uludağ(Bursa)	40°11′	29°04′	13.6	646	29.4	21	11
80	*A*. *nordmanniana*	TRK	Maçka(Trabzon)	41°00′	39°43°	14.1	874	25.6	41	9
95	*A*. *cephalonica*	GR	Kalamata	37°03′	22°07′	17.2	764	30.6	4	8
98	*A*. *borisii-regis*	GR	Kastoria(Pindus)	39°52′	20°48′	10.5	1033	27.1	32	17

IT: Italy; SK: Slovakia; PL: Poland; GER: Gemany; AT: Austria; F: France; TRK: Turkey; GR: Greece; AMT: mean annual temperature; AP: mean annual precipitation; maxTWM: warmest month temperature; PdM: driest month precipitation; N: number of sampled trees

**Table 2:** Bioclimatic variables used for correlation analysis.

Variable-code	Name	Explanation
AMT	Annual mean temperature	
MTWQ	Mean temperature of warmest quarter	
MTCQ	Mean temperature of coldest quarter	
maxTWM	Maximum temperature of warmest month	
minTCM	Minimum temperature of coldest month	
TAR	Temperature annual range	maxTWM-minTCM
MTwQ	Mean temperature of wettest quarter	
MTdQ	Mean temperature of driest quarter	
MDR	Mean diurnal range	Mean of monthly (maxTemp-minTemp)
ISO	Isothermality	(MDR/TAR)*100
TS	Temperature seasonality	Standard deviation*100
AP	Annual precipitation	
PwM	Precipitation of wettest month	
PdM	Precipitation of driest month	
PS	Precipitation seasonality	Coefficient of variation
PwQ	Precipitaton of wettest quarter	
PdQ	Precipitation of driest quarter	
PWQ	Precipitation of warmest quarter	
PCQ	Precipitation of coldest quarter	

**Table 3:** ANOVA results for wood properties and drought response measures for inter- and intra-specific data, respectively.

		1nter-specific				Intra-specific			
		Mean (SD)	df	F	*p*	Mean (SD)	df	*F*	*p*
	RW	2.462(0.802)		0.858	0.515	2.178(0.870)		2.335	0.021*
	EW	1.838(0.690)		0.782	0.567	1.568(0.736)		2.324	0.021*
	LW	0.625(0.179)		1.496	0.206	0.611(0.188)		3.404	0.001**
	RD	568.16(61.53)		2.068	0.083	601.74(61.84)		1.869	0.067
WP_coreage_	ED	434.62(41.51)		1.360	0.253	450.20(43.88)		1.563	0.138
	LD	916.61 (59.25)		3.450	0.009**	943.90(53.60)		1.437	0.184
	M1ND	302.19(41.85)		2.099	0.079	316.09(48.44)		1.432	0.186
	MAXD	1105.26(61.86)		3.085	0.016*	1125.81(56.27)		1.261	0.269
	LWP	27.74(7.56)		1.600	0.175	30.87(7.59)		3.238	0.001**
	Res	0.748(0.134)		1.861	0.118	0.718(0.161)		6.767	0.000***
DR_1986_	Rec	1.340(0.293)		0.947	0.459	1.350(0.333)		2.890	0.005**
	Rsl	0.992 (0.232)		2.240	0.065	0.947 (0.227)		2.721	0.008**
	rRsl	0.244(0.195)		1.322	0.27	0.229(0.191)		1.756	0.09
	Res	0.870(0.198)		1.676	0.158	1.019(0.220)		1.401	0.202
DR_1990_	Rec	1.064(0.170)		0.769	0.577	0.985(0.173)		2.513	0.014*
	Rsl	0.918(0.241)		1.061	0.393	1.004(0.282)		2.606	0.011*
	rRsl	0.049(0.147)	5	0.847	0.523	−0.015(0.175)	9	2.261	0.026*
	Res	0.675(0.183)		2.080	0.083	0.659(0.176)		2.640	0.010*
DR_1993_	Rec	1.371(0.362)		2.919	0.022*	1.460(0.359)		2.955	0.005**
	Rsl	0.888(0.226)		0.611	0.692	0.927 (0.227)		1.928	0.06
	rRsl	0.213(0.186)		2.442	0.047*	0.268(0.176)		1.550	0.146
	Res	0.526(0.130)		2.226	0.067	0.546(0.123)		2.041	0.045*
DR_2000_	Rec	1.537(0.313)		3.756	0.006**	1.614(0.283)		1.749	0.092
	Rsl	0.790(0.185)		0.523	0.757	0.865(0.174)		1.627	0.122
	rRsl	0.264(0.141)		2.435	0.047*	0.319(0.139)		1.620	0.124
	Res	0.574(0.112)		2.499	0.043*	0.553(0.100)		1.053	0.407
DR_2003_	Rec	1.571(0.429)		4.902	0.000***	1.636(0.478)		1.903	0.064
	Rsl	0.878(0.226)		5.062	0.000***	0.883(0.221)		1.335	0.233
	rRsl	0.303(0.235)		5.186	0.000***	0.330(0.222)		1.776	0.086
	Res	0.673(0.180)		11.560	0.000***	0.600(0.152)		1.273	0.265
DR_2007_	Rec	1.598(0.387)		8.862	0.000***	1.601(0.403)		1.514	0.158
	Rsl	1.027(0.205)		0.495	0.779	0.925(0.200)		1.725	0.097
	rRsl	0.355(0.189)		4.578	0.002**	0.325(0.200)		1.363	0.219

RW, EW, LW in mm, RD, ED, LD in kg/m^3^, LWP in %, df: degrees of freedom; significance levels.

* 0.05.

** 0.01.

*** 0.001.
