# Supplementary figures and images for: Inter- and intra-specific variation in drought sensitivity in Abies spec. and its relation to wood density and growth traits
Source: Agric For Meteorol. Author manuscript; Available in PMC 2016 Oct 4. (PMC5049588; doi:10.1016/j.agrformet.2015.08.268)

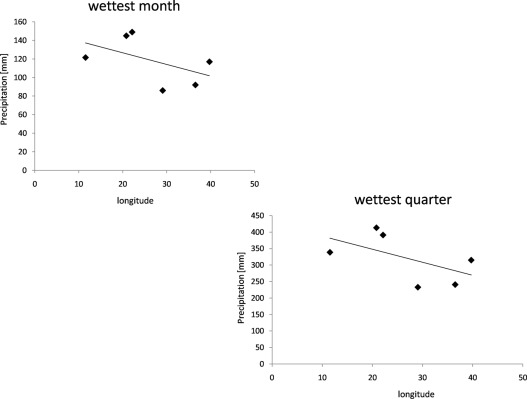

Supplement: Figure S1 [file NIHMS66319-supplement-Figure_S1.jpg]
